# Pricing decisions of risk averse logistic companies with carbon cap and trade under Stackelberg game

**DOI:** 10.1371/journal.pone.0287982

**Published:** 2023-07-19

**Authors:** Fuyou Huang, Bin Liu, Baoquan Tao, Yuankang Deng, Chao Ma

**Affiliations:** 1 Institute of Transportation Development Strategy & Planning of Sichuan Province, Chengdu, China; 2 Hubei Institute of Logistics Technology, Xiangyang, China; 3 Hubei Key Laboratory of Power System Design and Test for Electrical Vehicle, Hubei University of Arts and Science, Xiangyang, China; 4 School of Automobile and Traffic Engineering, Hubei University of Arts and Sciences, Xiangyang, China; Libyan Academy, LIBYA

## Abstract

With the implementation of the double carbon plan, this paper considers the delivery fees of two risk averse logistics companies under carbon cap and trade mechanism. We establish logistics company Stackelberg (MS) model and retailer Stackelberg (RS) model under mean variance (MV) framework, respectively. We obtain the optimal delivery fees and retail prices. We find out that the higher degree of risk aversion can lead to a lower delivery fee. We also show that a higher carbon trading price or a higher cross price sensitivity will increase delivery fees. Moreover, we indicate that the performances of logistics companies under MS scenario are higher than that RS scenario. In addition, we suggest that under the carbon cap and trade rules, in order to obtain higher profits, logistics companies should use fuel vehicle for transportation under certain conditions, and use electric vehicle in other cases.

## 1 Introduction

The carbon emissions from transportation industry account for 10.7% of the total global carbon emissions [1], and the carbon emissions from road transportation account for far more than 50% of the carbon emissions from transportation. In the past few decades, China’s transportation sector has 800 million tons of carbon dioxide, 60% of which comes from diesel emissions. In this case, it is very necessary to promote the electrification of freight transport. In fact, the transformation of freight vehicles to full electrification and the reduction of truck carbon emissions are the key tasks to achieve the goal of carbon neutrality.

In order to accelerate the implementation of carbon emission reduction, in July of 2021, China’s carbon emission market was launched for trading. In fact, before that, carbon trading had existed in China’s transportation sector. From 2007 to 2011, the first carbon trading amount of Chongqing BRT company was more than 4.6 million yuan. In 2016, Beijing Liquefied Natural Gas public transport project has been successfully sold in the pilot carbon trading market in Beijing. In 2018, the carbon inclusive emission reduction trading agreement of mobike bike (now renamed as “meituan bike”) was signed. It can be seen that under the goals of carbon peak and carbon neutralization, road freight transportation will gradually be included in the management of carbon trading system in the future. Which also urges freight companies to prepare in advance and actively carry out carbon reduction under the carbon trading mechanism.

As the main body of cargo transportation, freight companies can consider using electric vehicle (EV) for transportation under the guidance of carbon emission limits and trade regulations. At the same time, many cities in China will continue to provide subsidies for the distribution of electric commercial vehicles in 2023. In fact, several cities in China have introduced new energy freight subsidy policies, such as Foshan (http://jtys.foshan.gov.cn/gkmlpt/content/5/5211/mpost5211562.html), Taizhou (http://www.zjtz.gov.cn/art/2021/7/14/art12294298721643201.html), Nantong (http://jtysj.nantong.gov.cn//ntjy/zcjd1/content/34a1d851-27ee-4e46-90d1-3dcef995ae14.html). However, with the increasing uncertainty of today’s society, logistics companies may show a certain degree of risk aversion [2, 3]. So, how do risk-averse freight companies price distribution charges?

In addition, there are various logistics companies and retail companies of different sizes in the society. At this time, in the process of material transportation, there will be the phenomenon of “big store bully”. That is, in the transaction of material distribution, the company with strong strength will lead the weaker party. As the Stackelberg game can better describe the impact of the companies’ position on their decisions and profits in transaction process, such as [4–6]. So, when the logistics company is in different trading positions, how to determine the delivery fees?

To answer the questions mentioned earlier, we consider a basic model that includes two risk averse logistics companies and a risk neutral retailer. Under the carbon cap and trade mechanism, one logistics company uses fuel vehicle (FV) and the other uses EV in the distribution process. Expected utility (EU) [7], mean variance (MV) and conditional value at risk (CVaR) [8] are three common measures of risk aversion. Compared with EU and CVaR methods, MV has advantages in modeling risk aversion because it can transform random problems into deterministic approximate problems [9]. Therefore, we consider the MV method for modeling. Through the modeling under MV framework, we establish two kinds of Stackelberg game models under linear demand. Our main contributions are summarized as follows.

First, we consider the logistics company-lead model and retailer-lead model, respectively. We obtain the corresponding optimal delivery fees and retail prices.

Second, we discuss the parameter sensitivity. We show that the higher of companies’ risk aversion promotes the decrease of the companies’ delivery fees. We also point out that the higher carbon trading price leads to the higher delivery fees. Moreover, we reflect that a higher product substitutability leads to a higher delivery fee.

At last, we investigate the effects of power structure and the difference between the company using FV and company using EV. We indicate that the price decisions and profits of logistics companies in the leading position are higher than those in the following position. We also find out that the company using EV has a higher performance than company using FV under certain conditions.

The remainder of the paper is organized as follows. Next section reviews the literatures. In Section 3, we introduce the model description. Section 4 analyzes the models. Section 5 shows parameter sensitivity. Section 6 investigates the numerical studies. Finally, Section 7 concludes and presents future research. All technical proofs are presented in S1 Appendix.

## 2 Literature review

In this section, the literature is reviewed regarding two research streams to highlight the contributions.

The first related references are the supply chain with carbon cap and trade regulation. Under this rule, scholars put forward very good opinions on the supply chain decision making from a multi perspective.

Wang et al. [10] established three differential game models under the carbon cap and trade regulation, and showed that coop program can make more profits to firms than the non-coop scenario. Tang and Yang [11] analyzed the financing mechanism under carbon cap and trade, and pointed out that early repayment was better than bank loans. Wang et al. [12] investigated production and carbon emission reduction strategies under carbon cap and trade, they found that the implementation of cost-sharing contracts can increase carbon emission abatement level. Qi et al. [13] explored the optimal ordering and emission reduction level for risk-averse firm under carbon cap-and-trade, and pointed out that governments should continue to reduce carbon allowances as clean technologies evolve for the effectiveness of the carbon market. At the same time, scholars also considered the decision-making of reverse supply chain [14], dual channel supply chain [15], closed loop supply chain [16] and other models under carbon cap and trade. Moreover, some scholars have introduced low-carbon subsidies into the carbon cap and trade mechanism [17–19].

These series of achievements mentioned above provides us with a reference for us to make a decision of firms under carbon cap and trade mechanism from multiple scenarios. When the road freight transport company consider the carbon cap and trade rule, the existing literatures analyzed the impact of this policy on the firms [20] and propose an approach to carbon footprint management [21]. However, there is no discussion on the fees of road freight transport companies. Therefore, in contrast to the above works, we will study delivery fees considering carbon cap and trade.

The other related references are supply chain with behavioral preferences. Common behavioral preferences include fairness [22], loss aversion [23], and risk aversion [3]. We focus on the supply chain decision problem under risk aversion. In order to describe the risk aversion attitude of firms decision makers, MV is widely used in supply chain management. Chiu and Choi [24] reviewed a series of literature that focus on MV analytical models, which helped us better understand the MV model to manage the supply chain risk. He et al. [25] made the optimal decisions of channel members under carbon cap and trade policy through MV approach. Li et al. [26] studied manufacturer-led supply chain with carbon cap and trade by constructing mean-variance utility function, they pointed out that a low degree of risk avoidance will increase firms’ profits. Choi et al. [27] considered risk averse agents and retail competition under MV framework, and showed that the increasing or decreasing the retail selling price depends on the level of competition. Zhao et al. [28] discussed the contract strategy under MV method, and identified the conditions under which wholesale price contract and revenue sharing contract should be offered in a competitive market. Wang et al. [29] pointed out that both partners and consumers can benefit from the retailer’s risk control in the competitive dual-channel supply chain. These results strongly proved that MV method can better describe the risk aversion behavior of decision makers. We will continue to use the MV method to discuss the fees of logistics companies.

The differences between our study and the above are as follows. Although Yang and Yu [5] discussed the role of integrated logistics and procurement services provided by third-party logistics (3PL) in the Stackelberg game. Wang et al. [30] investigated the impact of 3PL’s risk aversion under MV framework. However, they ignored the carbon policy. Different from them, our problem is aimed at the risk averse logistics companies’ decisions in the supply chain with carbon policy. We mainly consider two logistics companies with different power vehicles, and describe the transportation costs of different power vehicles. On this basis, we describe the risk averse utilities of logistics companies under the guidance of carbon cap and trade policy under the MV framework. We determine the optimal delivery fees for maximizing utilities under two Stackelberg games.

## 3 Model description

This section describes the problem, gives the corresponding mathematical notation and meaning, and introduces basic models.

### 3.1 Problem description

**Fig 1:**
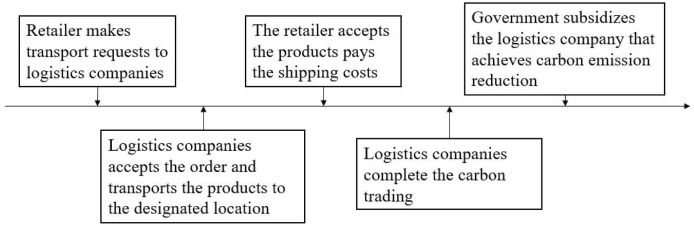
Transaction process.

We consider that retailer transport products through two competing logistics companies under random demand. One of the logistics companies uses FV (we called company FV, and denote it *M*_*o*_) for distribution and the other uses EV (we called company EV, and denote it *M*_*e*_). In order to encourage logistics companies to reduce carbon emissions, the government has set a carbon emissions ceiling, opened a carbon allowance trading market, and subsidized companies that achieve carbon emissions reductions. The transaction process under carbon cap and trade rule is shown in Fig 1. The retailer first proposes the transportation demand to the logistics companies, then the logistics companies accept the order, report the carbon emission data and complete the carbon trading. Subsequently the government subsidizes the logistics company that achieves carbon emission reduction, after that the logistics companies transport the products to the designated location, and finally the retailer accepts the products and pays the transportation cost.

### 3.2 Assumptions

To simplify the problem, we propose the following assumptions.

**Hypothesis 3.1**. *Both logistics companies only consider shipping heavy shipments and charge by weight*.
**Hypothesis 3.2**. *No loss of goods during transportation, labor and vehicle maintenance costs are negligible, also disregarding transaction time and transportation time*.
**Hypothesis 3.3**. *Logistics companies are honest and able to deliver the full product. The retailer is trustworthy and can pay the full cost of shipping. Carbon markets are efficient and able to execute carbon trading perfectly. The government is able to provide timely subsidies to logistics companies that reduce carbon emissions*.
**Hypothesis 3.4**. *Both logistics companies are risk averse and the retailer is risk neutral*.

### 3.3 Symbols and their meanings

For modeling the problem, the related notions are summarized in Table 1. Accordingly, the expectation, variance, and the standard deviation of the demand shock are *E*(*x*) = 0, *Var*(*x*) = *δ*^2^, and $\sqrt{Var(x)}=\delta$, respectively. For company *i*, the market demand is *D*_*i*_ = *a* − *bp*_*i*_ + *rp*_*j*_ + *x*, where *a* > 0, *b* > *r* > 0. We denote *r* that the degree of substitution between the two companies. If *r* is equal to zero, both companies are perfectly differentiated. As *r* becomes larger, the companies become more substitutable. The difference *b* − *r* is inversely related to the degree of company substitutability between the two companies. That is the smaller the difference, the more substitutable the two companies, therefore the more potential price competition.

**Table 1:** The notions and parameters.

Symbol	Description
*p*_*i*_	The selling price of retailer for logistics company *i*, Yuan per product
*w*_*i*_	The delivery fee for logistics company *i*, Yuan/kg⋅km
*c*_*o*_	The fuel expense of FV, Yuan/L
*α*	The oil consumption of FV, L/kg⋅km
*Q*_*o*_	The weight of the FV itself, kg
*e*_*o*_	The carbon emission coefficient of FV, kg/L
*c*_*e*_	The electric charge of EV, Yuan/ kWh
*β*	The power consumption of EV, kWh/kg⋅km
*Q*_*e*_	The weight of the EV itself, kg
*m*	The weight of single product, kg
*d*	The transportation distance, km
$\bar{e}$	The carbon cap, kg
*c*_*n*_	Carbon trading price, Yuan/kg
λ	Subsidy for carbon emission reduction, Yuan/kg
*a*	The average demand
*b*	The price sensitivity factor
*r*	The cross price sensitivity factor
*x*	The demand shock, which is defined on [−*U*, *U*]

Constrained by carbon cap and trade regulation, logistics companies need to pay the excess carbon emissions with the carbon trading price of *c*_*n*_ Yuan/kg when exceeding the carbon cap $\bar{e}$ kg. When carbon emissions quota is lower than $\bar{e}$, with the permission of the government, companies can sell to carbon trading market by *c*_*n*_ Yuan/kg. When using EV, the government will subsidize logistics company according to the amount of carbon reduction. In May 2021, Guangzhou City of China issued a policy stating that the government will subsidize the company at 10 yuan/ton of carbon dioxide according to the amount of carbon reduction. See https://www.gz.gov.cn/gfxwj/qjgfxwj/hpq/qbm/content/post7277979.html. Thus, we continue this subsidy approach, we assume that a subsidy of λ Yuan will be given for every kilogram of carbon emission reduction.

### 3.4 Basic models

According to the previous questions and assumptions, we model the retailer’s profit as the follow

$$\begin{array}{c} \begin{array}{cc} \pi_{R} & =Sales\;revenue-Freight\;fees\\ & =\left(p_{o}-w_{o}md\right)\left(a-bp_{o}+rp_{e}+x\right)+\left(p_{e}-w_{e}md\right)\left(a-bp_{e}+rp_{o}+x\right). \end{array} \end{array}$$ (1)

Correspondingly, the profits of logistics company FV and logistics company EV are as follows

$$\begin{array}{c} \begin{array}{c} \pi_{M_{o}}=Freight\;income-Fuel\;cost-Carbon\;trading\;cost\\ \;\;\;\;\;=w_{o}md\left(a-bp_{o}+rp_{e}+x\right)-c_{o}\alpha \left[m\left(a-bp_{o}+rp_{e}+x\right)+Q_{o}\right]d\\ \;\;\;\;\;\;\;\;-c_{n}\left\{e_{o}\alpha \left[m\left(a-bp_{o}+rp_{e}+x\right)+Q_{o}\right]d-\bar{e}\right\}. \end{array} \end{array}$$ (2)

$$\begin{array}{c} \begin{array}{c} \pi_{M_{e}}=Freight\;income-Electricity\;cost+Carbon\;trading\;income+Subsidy\\ \;\;\;\;\;=w_{e}md\left(a-bp_{e}+rp_{o}+x\right)-c_{e}\beta \left[m\left(a-bp_{e}+rp_{o}+x\right)+Q_{e}\right]d\\ \;\;\;\;\;\;\;\;+c_{n}\bar{e}+\lambda e_{o}\alpha m\left(a-bp_{e}+rp_{o}+x\right)d. \end{array} \end{array}$$ (3)

Noting that the retailer is risk neutral, then the expected profit of the manufacturer is

$$\begin{array}{c} \begin{array}{c} E\left(\pi_{R}\right)=\left(p_{o}-w_{o}md\right)\left(a-bp_{o}+rp_{e}\right)+\left(p_{e}-w_{e}md\right)\left(a-bp_{e}+rp_{o}\right). \end{array} \end{array}$$ (4)

Noting that logistics companies are risk averse, we use the MV framework to carve out their utility functions along. In line with [31], MV is defined as

$$\begin{array}{c} \begin{array}{c} MV\left(\pi_{i}\right)=E\left(\pi_{i}\right)-k_{i}\sqrt{Var(\pi_{i})}\\ \;\;\;\;=E\left(\pi_{i}\right)-k_{i}\sqrt{E{\left[\left(\pi_{i}\right)-E\left(\pi_{i}\right)\right]}^{2}}, \end{array} \end{array}$$ (5)

where *π*_*i*_ is the profit function, *E*(*π*_*i*_) and *Var*(*π*_*i*_) respect the mean and variance, respectively. *k*_*i*_ ≥ 0 is the risk aversion parameter. $\sqrt{Var(\pi_{i})}$ is the standard deviation, *MV*(*π*_*i*_) is the mean-standard-deviation objective function.

Then the MV function of logistic company FV and logistic company EV are as follows

$$\begin{array}{c} \begin{array}{c} MV\left(\pi_{M_{o}}\right)=E\left(\pi_{M_{o}}\right)-k_{o}\sqrt{E{\left[\pi_{M_{o}}-E\left(\pi_{M_{o}}\right)\right]}^{2}}\\ \;\;\;\;=\left[w_{o}-\left(c_{o}+c_{n}e_{o}\right)\alpha \right]\left(a-bp_{o}+rp_{e}-k_{o}\delta \right)md-\left(c_{o}+c_{n}e_{o}\right)\alpha dQ_{o}+c_{n}\bar{e}. \end{array} \end{array}$$ (6)

$$\begin{array}{c} \begin{array}{c} MV\left(\pi_{M_{e}}\right)=E\left(\pi_{M_{e}}\right)-k_{e}\sqrt{E{\left[\pi_{M_{e}}-E\left(\pi_{M_{e}}\right)\right]}^{2}}\\ \;\;\;\;=\left(w_{e}-c_{e}\beta +\lambda \alpha e_{o}\right)\left(a-bp_{e}+rp_{o}-k_{e}\delta \right)md-c_{e}\beta Q_{e}d+c_{n}\bar{e}. \end{array} \end{array}$$ (7)

## 4 Model analysis

**Fig 2:**
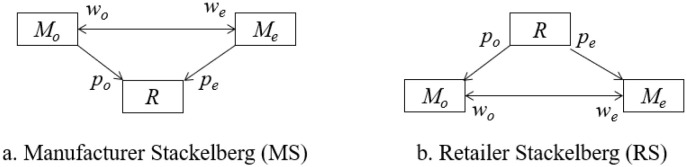
Game rules.

This section considers two possible power balance scenarios for which the rules of the game are given as the Fig 2. The first scenario is that the logistics companies act the leader and the retailer is the follower. The seconde scenario is that the retailer plays a leading role and the logistics companies act the followers.

### 4.1 Logistics company Stackelberg(MS)

Under the assumption MS, the logistics companies act the leader and take the retailer’s reaction function into consideration for their respective price decisions. The retailer’s reaction function given delivery fees *w*_*o*_ and *w*_*e*_ can be derived from the first-order conditions of Eq (4):

$$\begin{array}{c} \left\{ \begin{array}{c} \frac{\partial E(\pi_{R})}{\partial p_{o}}=a-2bp_{o}+2rp_{e}+\left(bw_{o}-rw_{e}\right)md=0,\\ \frac{\partial E(\pi_{R})}{\partial p_{e}}=a-2bp_{e}+2rp_{o}+\left(bw_{e}-rw_{o}\right)md=0. \end{array}\right. \end{array}$$ (8)

That is

$$\begin{array}{c} \left\{ \begin{array}{c} p_{o}=\frac{a+\left(b-r\right)w_{o}md}{2(b-r)},\\ p_{e}=\frac{a+\left(b-r\right)w_{e}md}{2(b-r)}. \end{array}\right. \end{array}$$ (9)

Taking the reaction functions Eq (9) into Eqs (6) and (7), respectively. We can obtain $MV\left(\pi_{M_{o}}\right)\left(p_{o}\left(w_{o}\right)\right)$ and $MV\left(\pi_{M_{e}}\right)\left(p_{e}\left(w_{e}\right)\right)$ as follows

$$\begin{array}{c} \begin{array}{c} MV\left(\pi_{M_{o}}\right)\left(p_{o}\left(w_{o}\right)\right)=\frac{1}{2}md\left[a-2\delta k_{o}+md\left(rw_{e}-bw_{o}\right)\right]\left[w_{o}-\alpha \left(c_{o}+c_{n}e_{o}\right)\right]\\ +c_{n}\bar{e}-\alpha d\left(c_{o}+c_{n}e_{o}\right)Q_{o}. \end{array} \end{array}$$ (10)

$$\begin{array}{c} \begin{array}{c} MV\left(\pi_{M_{e}}\right)\left(p_{e}\left(w_{e}\right)\right)=\frac{1}{2}md\left[a-2\delta k_{e}+md\left(rw_{o}-bw_{e}\right)\right]\left(w_{e}+\alpha \lambda e_{o}-\beta c_{e}\right)\\ +c_{n}\bar{e}-\beta dc_{e}Q_{e}. \end{array} \end{array}$$ (11)

Then the Nash equilibrium delivery fees can be derived from the first-order conditions of $MV\left(\pi_{M_{o}}\right)\left(p_{o}\left(w_{o}\right)\right)$ and $MV\left(\pi_{M_{e}}\right)\left(p_{e}\left(w_{e}\right)\right)$. That is

$$\begin{array}{c} w_{o}^{MS}=\frac{a}{(2b-r)md}+\frac{b\left[r\left(\beta c_{e}-\lambda \alpha e_{o}\right)+2b\alpha \left(c_{o}+c_{n}e_{o}\right)\right]-2\delta \left(rk_{e}+2bk_{o}\right)}{4b^{2}-r^{2}}. \end{array}$$ (12)

$$\begin{array}{c} w_{e}^{MS}=\frac{a}{(2b-r)md}+\frac{b\left[2b\left(\beta c_{e}-\lambda \alpha e_{o}\right)+r\alpha \left(c_{o}+c_{n}e_{o}\right)\right]-2\delta \left(rk_{o}+2bk_{e}\right)}{4b^{2}-r^{2}}. \end{array}$$ (13)

Thus the corresponding retail prices can be obtained via substituting Eqs (12) and (13) into Eq (9):

$$\begin{array}{c} p_{o}^{MS}=\frac{a(3b-2r)}{2(b-r)(2b-r)}+\frac{bmd\left[r\left(\beta c_{e}-\lambda \alpha e_{o}\right)+2b\alpha \left(c_{o}+c_{n}e_{o}\right)\right]-2\delta \left(rk_{e}+2bk_{o}\right)}{2(4b^{2}-r^{2})}. \end{array}$$ (14)

$$\begin{array}{c} p_{e}^{MS}=\frac{a(3b-2r)}{2(b-r)(2b-r)}+\frac{bmd\left[2b\left(\beta c_{e}-\lambda \alpha e_{o}\right)+r\alpha \left(c_{o}+c_{n}e_{o}\right)\right]-2\delta \left(rk_{o}+2bk_{e}\right)}{2(4b^{2}-r^{2})}. \end{array}$$ (15)

### 4.2 Retailer Stackelberg(RS)

Under the assumption RS, the retailer becomes the leader and the logistics companies as the followers. The retailer takes the logistics companies’ reaction functions into account for its own retail price decisions.

Let *p*_*o*_ = *l*_*o*_ + *w*_*o*_*md* and *p*_*e*_ = *l*_*e*_ + *w*_*e*_*md*, we can rewrite the Eqs (6) and (7) as

$$\begin{array}{c} \begin{array}{c} MV\left(\pi_{M_{o}}\left(w_{o}\right)\right)=\left[w_{o}-\left(c_{o}+c_{n}e_{o}\right)\alpha \right]\left[a-b\left(w_{o}md+l_{o}\right)+rp_{e}-k_{o}\delta \right]md\\ \;\;\;\;\;\;\;\;\;\;\;\;\;\;\;\;\;\;\;\;\;\;\;\;-\left(c_{o}+c_{n}e_{o}\right)\alpha dQ_{o}+c_{n}\bar{e}. \end{array} \end{array}$$ (16)

$$\begin{array}{c} \begin{array}{c} MV\left(\pi_{M_{e}}\left(w_{e}\right)\right)=\left(w_{e}-c_{e}+\lambda \alpha e_{o}\right)\left[a-b\left(w_{e}md+l_{e}\right)+rp_{o}-k_{e}\delta \right]md\\ \;\;\;\;\;\;\;\;\;\;\;\;\;\;\;\;\;\;\;\;\;\;\;\;-c_{e}\beta Q_{e}d+c_{n}\bar{e}. \end{array} \end{array}$$ (17)

Thus the reaction functions of logistics companies can be derived from the following first-order conditions:

$$\begin{array}{c} \left\{ \begin{array}{c} \frac{\partial MV(\pi_{M_{o}}\left(w_{o}\right))}{\partial w_{o}}=\left[a+bmd\left(\alpha c_{o}+\alpha c_{n}e_{o}-2w_{o}\right)+rp_{e}-\delta k_{o}-bl_{o}\right]md=0,\\ \frac{\partial MV(\pi_{M_{e}}\left(w_{e}\right))}{\partial w_{e}}=\left[a+bmd\left(\beta c_{e}-\alpha \lambda e_{o}-2w_{e}\right)+rp_{o}-\delta k_{e}-bl_{e}\right]md=0. \end{array}\right. \end{array}$$ (18)

Combining *l*_*o*_ = *p*_*o*_ − *w*_*o*_*md* and *l*_*e*_ = *p*_*e*_ − *w*_*e*_*md*, the retailer’s reaction functions can be derived from Eq (18):

$$\begin{array}{c} \left\{ \begin{array}{c} w_{o}=\frac{a+bmd\left(\alpha c_{o}+\alpha c_{n}e_{o}\right)+rp_{e}-\delta k_{o}-bp_{o}}{bmd},\\ w_{e}=\frac{a+bmd\left(\beta c_{e}-\alpha \lambda e_{o}\right)+rp_{o}-\delta k_{e}-bp_{e}}{bmd}. \end{array}\right. \end{array}$$ (19)

Taking the reaction functions Eq (19) into Eq (4), we can obtain *E*(*π*_*R*_)(*p*_*o*_, *p*_*e*_) as follows

$$\begin{array}{c} \left\{ \begin{array}{c} E\left(\pi_{R}\right)\left(p_{o},p_{e}\right)=\frac{\left(2bp_{o}-a-bmd\left(\alpha c_{o}+\alpha c_{n}e_{o}\right)-rp_{e}+\delta k_{o}\right)\left(a-bp_{o}+rp_{e}\right)}{b}\\ +\frac{\left(2bp_{e}-a-bmd\left(\beta c_{e}-\alpha \lambda e_{o}\right)-rp_{o}+\delta k_{e}\right)\left(a-bp_{e}+rp_{o}\right)}{b} \end{array}\right. \end{array}$$ (20)

By solving the first-order conditions $\frac{\partial E\left(\pi_{R}\right)\left(p_{o},p_{e}\right)}{\partial p_{o}}=0$ and $\frac{\partial E\left(\pi_{R}\right)\left(p_{o},p_{e}\right)}{\partial p_{e}}=0$. Then the optimal retail prices are as the following

$$\begin{array}{c} p_{o}^{RS}=\frac{a(6b^{2}-br-2r^{2})}{2\left(b-r\right)\left(4b^{2}-r^{2}\right)}+\frac{bmd\left[r\left(\beta c_{e}-\alpha \lambda e_{o}\right)+2b\alpha \left(c_{o}+c_{n}e_{o}\right)\right]-\delta \left(rk_{e}+2bk_{o}\right)}{2(4b^{2}-r^{2})}. \end{array}$$ (21)

$$\begin{array}{c} p_{e}^{RS}=\frac{a(6b^{2}-br-2r^{2})}{2\left(b-r\right)\left(4b^{2}-r^{2}\right)}+\frac{bmd\left[2b\left(\beta c_{e}-\alpha \lambda e_{o}\right)+r\alpha \left(c_{o}+c_{n}e_{o}\right)\right]-\delta \left(2bk_{e}+rk_{o}\right)}{2(4b^{2}-r^{2})}. \end{array}$$ (22)

Thus the corresponding delivery fees can be obtained via substituting Eqs (21) and (22) into Eq (19):

$$\begin{array}{c} w_{o}^{RS}=\frac{ab\left(2b+r\right)-\delta [brk_{e}+\left(6b^{2}-r^{2}\right)k_{o}]}{2bmd(4b^{2}-r^{2})}+\frac{br\left(\beta c_{e}-\lambda \alpha e_{o}\right)+\left(6b^{2}-r^{2}\right)\alpha \left(c_{o}+c_{n}e_{o}\right)}{2(4b^{2}-r^{2})}. \end{array}$$ (23)

$$\begin{array}{c} w_{e}^{RS}=\frac{ab\left(2b+r\right)-\delta [\left(6b^{2}-r^{2}\right)k_{e}+brk_{o}]}{2bmd(4b^{2}-r^{2})}+\frac{\left(6b^{2}-r^{2}\right)\left(\beta c_{e}-\lambda \alpha e_{o}\right)+br\alpha \left(c_{o}+c_{n}e_{o}\right)}{2(4b^{2}-r^{2})}. \end{array}$$ (24)

## 5 Sensitivity analysis

In this section, we discuss several implications of the results. Especially, we focus on the effects of logistics companies’ risk aversion, demand fluctuation, carbon trade price elasticity, product price differentiation on agents’ decisions, respectively.

**Proposition 5.1**. *The delivery fees and retail prices are decreasing in* *k*_*o*_ *and k*_*e*_, *respectively*.

Proposition 5.1 reflects the greater the risk aversion coefficient are, the lower the decisions are. That is to say, the more a logistics company is afraid of risks, the lower the freight fee for fear of losing customers. So the competitor will also lower the delivery fee. As a result, retailers will reduce the retail price with the reduction of transportation costs, and in exchange for more sales markets.

**Proposition 5.2**. *The delivery fees and retail prices are decreasing in δ*.

Proposition 5.2 reflects the greater the standard deviation of demand shock is, the lower the decisions are. That is to say, with the increase of demand fluctuation, the retailer tends to appropriately reduce the retail price in order to stabilize the market. At the same time, in order to ensure the stability of transportation share, it is a reasonable decision for logistics companies to reduce the freight properly.

**Proposition 5.3**. *The price decisions of agents are increasing in c*_*n*_.

Proposition 5.3 reflects the price decisions of agents’ increase with the increase of carbon trading price.This means that the increase of carbon trading price will lead to the increase of cost for logistics company FV. In order to alleviate the imbalance of income and expenditure dilemma, logistics company FV appropriately increases the distribution fee to ensure the matching of income and expenditure. At the same time, although the revenue of logistics company EV increases under the impact of carbon trading, the possibility of logistics company FV to increase the freight rate under carbon trading is considered. In order to protect the robustness of the transportation market, logistics company EV will increase the transportation price appropriately. As a result of the increase in freight rates for logistics companies, this also leads to an increase in retail prices for the retailer.

**Proposition 5.4**. *The price decisions of agents are decreasing in b. And the price decisions of agents are increasing in r*.

Proposition 5.4 reflects agents should appropriately reduce their prices as the price sensitivity coefficient of products increases, and appropriately increase their prices as the cross price sensitivity coefficient of products increases. This indicates that the higher the product substitutability, the higher the transportation price.

## 6 Numerical studies

To further discuss the effects of firms’ risk-averse behavior, the price of carbon trading and firms’ substitutability on firms’ decisions and performances, this section will work with numerical experiments. We observe the changes of retailer and logistics companies’ decisions and the performance changes of logistics companies by varying the size of key variables.

In the numerical investigations, we apply light truck car transaction data to investigate the significance of management aspects. We examined the two light trucks of FAW Jiefang. They are both 4.2 meters long and have a total vehicle load of 4.495 tons. Among them, the one burning diesel is “Jiefang tiger V”, and its parameters are *c*_*o*_ = 8.22 Yuan/L, *α* = 0.000034 L/kg⋅km, *Q*_*o*_ = 2805 kg,*e*_*o*_ = 2.7 kg/L. The other is “Jiefang J6F” electric vehicle and its parameters are *c*_*e*_ = 1 Yuan/kWh, *β* = 0.00016 kWh/kg⋅km, *Q*_*e*_ = 2995 kg. The other parameters are $\bar{e}=50$ kg, *a* = 1000, *b* = 1, *r* = 0.4, λ = 0.001 Yuan/kg, *c*_*n*_ = 0.0585 Yuan/kg, *m* = 0.5 kg, *d* = 150 km, *k*_*o*_ = 0.4, *k*_*e*_ = 0.7, *δ* = 1. In these numerical studies, we change one parameter and fixed the others.

In the following, we first consider the difference of prices and performances between using EV and FV for transportation.

Considering the impact of logistics companies’ risk aversion. Table 2 shows $w_{o}^{MS}>w_{e}^{MS}$ and $p_{o}^{MS}>p_{e}^{MS}$ if *k*_*o*_ < 1.00469, $w_{o}^{RS}>w_{e}^{RS}$ and $p_{o}^{RS}>p_{e}^{RS}$ if *k*_*o*_ < 1.00937. Table 3 shows $w_{o}^{MS}>w_{e}^{MS}$ and $p_{o}^{MS}>p_{e}^{MS}$ if *k*_*e*_ > 0.990629, $w_{o}^{RS}>w_{e}^{RS}$ and $p_{o}^{RS}>p_{e}^{RS}$ if *k*_*e*_ > 0.995315. According to Tables 2 and 3, it reflects that $w_{o}^{MS}>w_{e}^{MS}$ and $p_{o}^{MS}>p_{e}^{MS}$ always hold if *k*_*o*_ < *k*_*e*_. This indicates that compared with company FV, when company EV is more risk averse, the delivery fee and retail price of using EV for transportation are lower than that of using FV. In other words, compared with low risk aversion logistics companies, high risk aversion logistics companies have lower delivery fee decisions.

**Table 2:** Impact of *k*_*o*_ on agents’ decisions.

Decisions	MS	RS
*w*_*o*_	8.33072 − 0.0138889*k*_*o*_	4.1662 − 0.0101389*k*_*o*_
*w*_*e*_	8.31956 − 0.00277778*k*_*o*_	4.15666 − 0.000694444*k*_*o*_
*p*_*o*_	1145.74 − 0.520833*k*_*o*_	1145.79 − 0.260417*k*_*o*_
*p*_*e*_	1145.32 − 0.104167*k*_*o*_	1145.58 − 0.0520833*k*_*o*_

**Table 3:** Impact of *k*_*e*_ on agents’ decisions.

Decisions	MS	RS
*w*_*o*_	8.31961 − 0.00277778*k*_*e*_	4.15675 − 0.000694444*k*_*e*_
*w*_*e*_	8.33067 − 0.0138889*k*_*e*_	4.16611 − 0.0101389*k*_*e*_
*p*_*o*_	1145.32 − 0.104167*k*_*e*_	1145.58 − 0.0520833*k*_*e*_
*p*_*e*_	1145.73 − 0.520833*k*_*e*_	1145.79 − 0.260417*k*_*e*_

**Fig 3:**
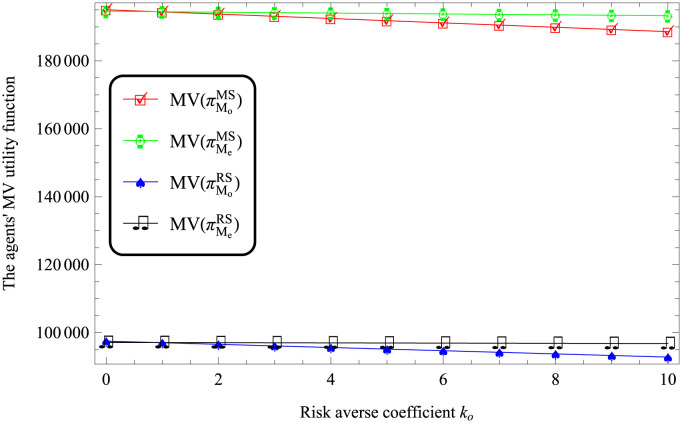
Impact of *k*_*o*_ on the logistics companies’ performances.

**Fig 4:**
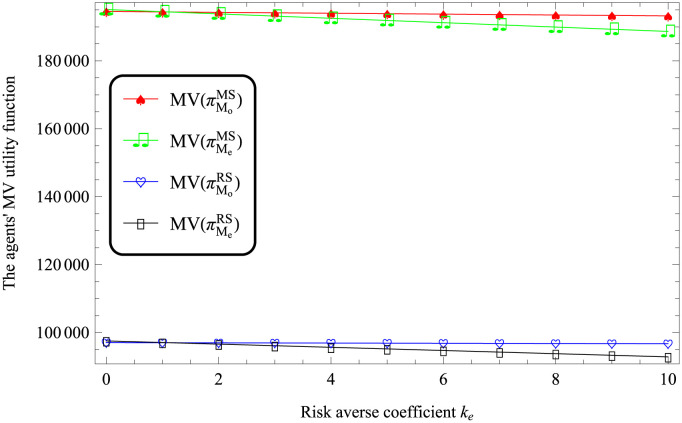
Impact of *k*_*e*_ on the logistics companies’ performances.

According to Figs 3 and 4, it always holds that $MV\left(\pi_{M_{e}}\right)>MV\left(\pi_{M_{o}}\right)$ under both MS and RS scenarios. That is, the utility of logistics company EV is more than that of logistics company FV.

**Fig 5:**
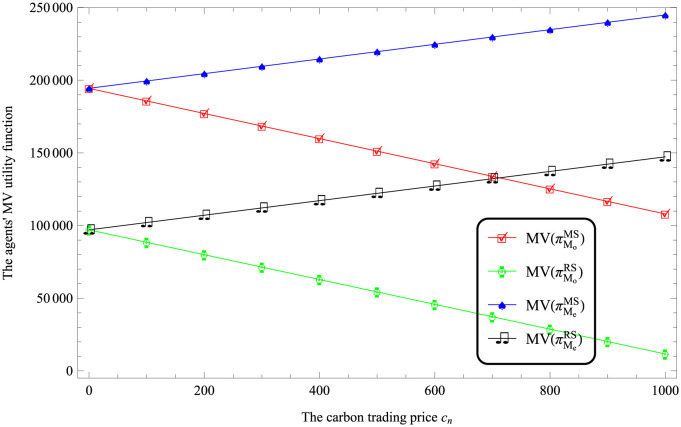
Impact of *c*_*n*_ on the logistics companies’ performances.

Considering the impact of carbon trading price. Table 4 shows *w*_*o*_ > *w*_*e*_ and *p*_*o*_ > *p*_*e*_ if *c*_*n*_ > −1.30253 under both MS and RS scenarios. This indicates that the price decision of using FV for transportation is higher than that of using EV when implementing carbon cap and trade policies. Fig 5 shows that $MV\left(\pi_{M_{e}}\right)>MV\left(\pi_{M_{o}}\right)$ under both MS and RS scenarios.

**Table 4:** Impact of *c*_*n*_ on agents’ decisions.

Decisions	MS	RS
*w*_*o*_	8.31683 + 4.78125 × 10^−5^*c*_*n*_	4.15605 + 6.98063 × 10^−5^*c*_*n*_
*w*_*e*_	8.31678 + 9.5625 × 10^−6^*c*_*n*_	4.15597 + 4.78125 × 10^−6^*c*_*n*_
*p*_*o*_	1145.21 + 1.79297 × 10^−3^*c*_*n*_	1145.53 + 1.79297 × 10^−3^*c*_*n*_
*p*_*e*_	1145.21 + 3.58594 × 10^−4^*c*_*n*_	1145.53 + 3.58594 × 10^−4^*c*_*n*_

Considering the impact of the cross price sensitivity factor. Table 5 shows *w*_*o*_ > *w*_*e*_ and *p*_*o*_ > *p*_*e*_ if 0 < *r* < 1 = *b* under both MS and RS scenarios. This indicates that no matter how different the product price is, the price decision of using FV for transportation is higher than that of using EV. In addition, via numerical studies, $MV\left(\pi_{M_{e}}\right)>MV\left(\pi_{M_{o}}\right)$ if 0 < *r* < 1 = *b* under both MS and RS scenarios.

**Table 5:** Impact of *c*_*n*_ on agents’ decisions.

Decisions	MS	RS
*w*_*o*_	$\frac{1996.04+998.005r}{300-75r^{2}}$	$\frac{1994.13+999.005r+0.978636r^{2}}{600-150r^{2}}$
*w*_*e*_	$\frac{1996.01+998.021r}{300-75r^{2}}$	$\frac{13.2935+6.66014r+0.00663257r^{2}}{4-r^{2}}$
*p*_*o*_	$\frac{2998.02-499.019r-999.003r^{2}}{\left(4-r^{2}\right)\left(r-1\right)}$	$\frac{2999.02-499.519r-999.503r^{2}}{4-4r-r^{2}+r^{3}}$
*p*_*e*_	$\frac{2998.01-498.994r-999.011r^{2}}{\left(4-r^{2}\right)\left(r-1\right)}$	$\frac{2999.01-499.494r-999.511r^{2}}{4-4r-r^{2}+r^{3}}$

**Fig 6:**
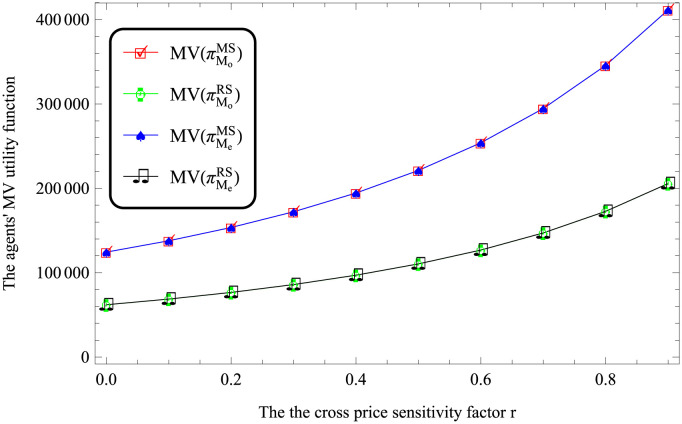
Impact of *r* on the logistics companies’ performances.

According to Figures from Figs 3 to 6, the utility of logistics companies using FV for transportation is lower than that of logistics companies using EV.

We then discuss the effects of power structures on decisions and logistics companies’ performances. We obtain the following results.

**Proposition 6.1**. $w_{i}^{RS}<w_{i}^{MS}$ *and* $p_{i}^{RS}>p_{i}^{MS}$, *where i respect o and e*.

Proposition 6.1 reflects that the prices determined when the agents are in the leading position are higher than when they are in the following position. This further indicates that companies with greater power have more voice.

According to Figures from Figs 3 to 6, it reflects that $MV\left(\pi_{M_{i}}^{MS}\right)>MV\left(\pi_{M_{i}}^{RS}\right)$, where *i* respect *o* and *e*. This indicates that compared with the retailer-dominated model, the logistics companies in the dominant position have higher utilities.

## 7 Conclusions

With the implementation of the dual carbon policy, the carbon trading market has been officially launched in China. Under the carbon cap and trade regulation, how should road transport companies charge freight to maximize benefits? Nowadays, the goods distribution market is uncertain, and freight companies often take a risk averse attitude. At this time, how do they make decisions? Based on this, this paper considers a supply chain composed of a freight company using FV, a freight company using EV and a retailer. We discuss the optimal delivery fees under carbon cap and trade by using the mean variance approach under logistics company Stackelberg game and retailer Stackelberg game, respectively. The results are as follows:

First, we show that a higher risk aversion coefficient lead to a lower delivery fee and retail price. Second, we find that a higher standard deviation of demand shock leads to a lower delivery fees. Third, we verify that the higher the carbon trade price, the higher the freight fees charge charged by companies. Fourth, we demonstrate that the increase of consumers’ sensitivity to product prices will lead to a decline in delivery fees. While the increase of sensitivity to product cross price will promote the increase of delivery fees. Moreover, we point out that the prices determined and performances obtained when the agents are in the leading position are higher than when they are in the following position. At last, we reveal that logistics companies using EV can obtain more performance than logistics companies using FV under certain conditions.

A managerial insight is that under the carbon cap and carbon trading mechanism, the behavior that the logistics companies replace FV with EV is very helpful to the companies’ revenue. This implies that under the carbon cap and trade rules, logistics companies can use EV to carry out their work in the short and medium distance transportation business. On the one hand, this can effectively increase the income of logistics companies themselves. On the other hand, it can respond to the national call for carbon peaking and carbon neutrality, and make their own contribution to the sustainable development of society. Therefore, it is suggested that under the condition of sufficient funds, road freight transport companies should speed up the use of new energy vehicles for cargo distribution.

This study has several limitations. Average fuel consumption (energy consumption) is affected by traffic conditions, this is what we have not considered. For further research, we can consider the issue of pricing when traffic jam is involved. Our article only considers charging according to the weight of the goods, but we have not considered the distribution of measurement cargo. Therefore, in another direction, we can discuss the decision-making when charging by volume.

## Supporting information

S1 Appendix (PDF)
