# Supplementary material for: Pricing decisions of risk averse logistic companies with carbon cap and trade under Stackelberg game
Source: PLoS One. 2023 Jul 19;18(7):e0287982. doi: 10.1371/journal.pone.0287982 (PMC10355466; doi:10.1371/journal.pone.0287982)
Supplement: S1 Appendix — (PDF) [file pone.0287982.s001.pdf]

Appendixs

Proof of the optimal decisions under MS scenario.

Recalling that the expected profit of the manufacturer is

$$E(\pi_R) = (p_o - w_o md)(a - bp_o + rp_e) + (p_e - w_e md)(a - bp_e + rp_o). \tag{1}$$

It holds that $\frac{\partial^2 E(\pi_R)}{p_o^2} = -2b < 0$, and

$$\begin{vmatrix} \frac{\partial^2 E(\pi_R)}{\partial p_o^2} & \frac{\partial^2 E(\pi_R)}{\partial p_o \partial p_e} \\ \frac{\partial^2 E(\pi_R)}{\partial p_e \partial p_o} & \frac{\partial^2 E(\pi_R)}{\partial p_e^2} \end{vmatrix} = 4(b^2 - r^2) > 0.$$

Then Hessian matrix is negative-definite. Thus, we can obtain the retailer's reaction functions as follows

$$\begin{cases} p_o = \frac{a+(b-r)w_o md}{2(b-r)}, \\ p_e = \frac{a+(b-r)w_e}{2(b-r)}. \end{cases} \tag{2}$$

Noting that the MV function of logistic company FV and logistic company EV are as follows

$$\begin{aligned} MV(\pi_{M_o}) &= E(\pi_{M_o}) - k_o\sqrt{E[\pi_{M_o} - E(\pi_{M_o})]^2} \\ &= [w_o - (c_o + c_n e_o)\alpha](a - bp_o + rp_e - k_o\delta)md - (c_o + c_n e_o)\alpha d Q_o + c_n \bar{e}. \end{aligned} \tag{3}$$

$$\begin{aligned} MV(\pi_{M_e}) &= E(\pi_{M_e}) - k_e\sqrt{E[\pi_{M_e} - E(\pi_{M_e})]^2} \\ &= (w_e - c_e + \lambda\alpha e_o)(a - bp_e + rp_o - k_e\delta)md - c_e\beta Q_e d + c_n \bar{e}. \end{aligned} \tag{4}$$

Then taking Eq. (2) into Eqs. (11) and (12), respectively. We can obtain that

$$MV(\pi_{M_o})(p_o(w_o)) = \frac{1}{2}md[a - 2\delta k_o + md(rw_e - bw_o)][w_o - \alpha(c_o + c_n e_o)] + c_n\bar{e} - \alpha d(c_o + c_n e_o)Q_o. \tag{5}$$

$$MV(\pi_{M_e})(p_e(w_e)) = \frac{1}{2}md[a - 2\delta k_e + md(rw_o - bw_e)](w_e + \alpha\lambda e_o - \beta c_e) + c_n\bar{e} - \beta d c_e Q_e. \tag{6}$$

Since $\frac{\partial^2 MV(\pi_{M_o})(p_o(w_o))}{w_o^2} = -bm^2d^2 < 0$, and

$$\begin{vmatrix} \frac{\partial^2 MV(\pi_{M_o})(p_o(w_o))}{\partial w_o^2} & \frac{\partial^2 MV(\pi_{M_o})(p_o(w_o))}{\partial w_o \partial w_e} \\ \frac{\partial^2 MV(\pi_{M_o})(p_o(w_o))}{\partial w_e \partial w_o} & \frac{\partial^2 MV(\pi_{M_o})(p_o(w_o))}{\partial w_e^2} \end{vmatrix} = \frac{1}{4}m^4d^4(4b^2 - r^2) > 0.$$

Then Hessian matrix is negative-definite. Thus, we can obtain the optimal delivery fees are follows

$$w_o^{MS} = \frac{a}{(2b-r)md} + \frac{b[r(\beta c_e - \lambda\alpha e_o) + 2b\alpha(c_o + c_n e_o)] - 2\delta(rk_e + 2bk_o)}{4b^2 - r^2}. \tag{7}$$

$$w_e^{MS} = \frac{a}{(2b-r)md} + \frac{b[2b(\beta c_e - \lambda\alpha e_o) + r\alpha(c_o + c_n e_o)] - 2\delta(rk_o + 2bk_e)}{(4b^2 - r^2)}. \quad (8)$$

Then the retail prices can be obtained by substituting Eqs. (7) and (8) into Eq. (2):

$$p_o^{MS} = \frac{a(3b-2r)}{2(b-r)(2b-r)} + \frac{bmd[r(\beta c_e - \lambda\alpha e_o) + 2b\alpha(c_o + c_n e_o)] - 2\delta(rk_e + 2bk_o)}{2(4b^2 - r^2)}. \quad (9)$$

$$p_e^{MS} = \frac{a(3b-2r)}{2(b-r)(2b-r)} + \frac{bmd[2b(\beta c_e - \lambda\alpha e_o) + r\alpha(c_o + c_n e_o)] - 2\delta(rk_o + 2bk_e)}{2(4b^2 - r^2)}. \quad (10)$$

Proof of the optimal decisions under RS scenario.

Recalling that the MV function of logistic company FV and logistic company EV

$$\begin{aligned} MV(\pi_{M_o}) &= E(\pi_{M_o}) - k_o\sqrt{E[\pi_{M_o} - E(\pi_{M_o})]^2} \\ &= [w_o - (c_o + c_n e_o)\alpha](a - bp_o + rp_e - k_o\delta)md - (c_o + c_n e_o)\alpha d Q_o + c_n\bar{e}. \end{aligned} \quad (11)$$

$$\begin{aligned} MV(\pi_{M_e}) &= E(\pi_{M_e}) - k_e\sqrt{E[\pi_{M_e} - E(\pi_{M_e})]^2} \\ &= (w_e - c_e + \lambda\alpha e_o)(a - bp_e + rp_o - k_e\delta)md - c_e\beta Q_e d + c_n\bar{e}. \end{aligned} \quad (12)$$

.

Let $p_o = l_o + w_o md$ and $p_e = l_e + w_e md$, we can rewrite the Eqs. (11) and (12) as

$$\begin{aligned} MV(\pi_{M_o}(w_o)) = [w_o - (c_o + c_n e_o)\alpha][a - b(w_o md + l_o) + rp_e - k_o\delta]md \\ - (c_o + c_n e_o)\alpha d Q_o + c_n\bar{e}. \end{aligned} \quad (13)$$

$$\begin{aligned} MV(\pi_{M_e}(w_e)) = (w_e - c_e + \lambda\alpha e_o)[a - b(w_e md + l_e) + rp_o - k_e\delta]md \\ - c_e\beta Q_e d + c_n\bar{e}. \end{aligned} \quad (14)$$

Since $\frac{\partial^2 MV(\pi_{M_o}(w_o))}{\partial w_o^2} = -2bm^2d^2$, and

$$\begin{vmatrix} \frac{\partial^2 MV(\pi_{M_o}(w_o))}{\partial w_o^2} & \frac{\partial^2 MV(\pi_{M_o}(w_o))}{\partial w_o \partial w_e} \\ \frac{\partial^2 MV(\pi_{M_e}(w_e))}{\partial w_e \partial w_o} & \frac{\partial^2 MV(\pi_{M_e}(w_e))}{\partial w_e^2} \end{vmatrix} = 4b^2m^4d^4 > 0.$$

Then Hessian matrix is negative-definite. Thus, we can obtain that the logistic companies' reaction functions as the following

$$\begin{cases} \frac{\partial MV(\pi_{M_o}(w_o))}{\partial w_o} = [a + bmd(\alpha c_o + \alpha c_n e_o - 2w_o) + rp_e - \delta k_o - bl_o]md = 0, \\ \frac{\partial MV(\pi_{M_e}(w_e))}{\partial w_e} = [a + bmd(\beta c_e - \alpha\lambda e_o - 2w_e) + rp_o - \delta k_e - bl_e]md = 0. \end{cases} \quad (15)$$

Combining $l_o = p_o - w_o md$ and $l_e = p_e - w_e md$, then the retailer's reaction functions can be derived from Eq. (15):

$$\begin{cases} w_o = \frac{a + bmd(\alpha c_o + \alpha c_n e_o) + rp_e - \delta k_o - bp_o}{bmd}, \\ w_e = \frac{a + bmd(\beta c_e - \alpha\lambda e_o) + rp_o - \delta k_e - bp_e}{bmd}. \end{cases} \quad (16)$$

Taking the reaction functions Eq. (16) into Eq. (1), we can obtain $E(\pi_R)(p_o, p_e)$ as follows

$$\begin{cases} E(\pi_R)(p_o, p_e) = \frac{(2bp_o - a - bmd(\alpha c_o + \alpha c_n e_o) - rp_e + \delta k_o)(a - bp_o + rp_e)}{b} \\ \qquad + \frac{(2bp_e - a - bmd(\beta c_e - \alpha\lambda e_o) - rp_o + \delta k_e)(a - bp_e + rp_o)}{b} \end{cases} \tag{17}$$

Since $\frac{\partial^2 E(\pi_R)(p_o,p_e)}{p_o^2} = -4b - \frac{2r^2}{b} < 0$, and

$$\begin{vmatrix} \frac{\partial^2 E(\pi_R)(p_o,p_e)}{\partial p_o^2} & \frac{\partial^2 E(\pi_R)(p_o,p_e)}{\partial p_o \partial p_e} \\ \frac{\partial^2 E(\pi_R)(p_o,p_e)}{\partial p_e \partial p_o} & \frac{\partial^2 E(\pi_R)(p_o,p_e)}{\partial p_e^2} \end{vmatrix} = \frac{4(b^2 - r^2)(4b^2 - r^2)}{b^2} > 0.$$

Then Hessian matrix is negative-definite. Thus, the optimal retail prices satisfy the first-order conditions $\frac{\partial E(\pi_R)(p_o,p_e)}{\partial p_o} = 0$ and $\frac{\partial E(\pi_R)(p_o,p_e)}{\partial p_e} = 0$. That is

$$p_o^{RS} = \frac{a(6b^2 - br - 2r^2)}{2(b-r)(4b^2 - r^2)} + \frac{bmd[r(\beta c_e - \alpha\lambda e_o) + 2b\alpha(c_o + c_n e_o)] - \delta(rk_e + 2bk_o)}{2(4b^2 - r^2)}. \tag{18}$$

$$p_e^{RS} = \frac{a(6b^2 - br - 2r^2)}{2(b-r)(4b^2 - r^2)} + \frac{bmd[2b(\beta c_e - \alpha\lambda e_o) + r\alpha(c_o + c_n e_o)] - \delta(2bk_e + rk_o)}{2(4b^2 - r^2)}. \tag{19}$$

Then the delivery fees can be obtained by substituting Eqs. (18) and (19) into Eq. (16):

$$w_o^{RS} = \frac{ab(2b + r) - \delta[brk_e + (6b^2 - r^2)k_o]}{2bmd(4b^2 - r^2)} + \frac{br(\beta c_e - \lambda\alpha e_o) + (6b^2 - r^2)\alpha(c_o + c_n e_o)}{2(4b^2 - r^2)}. \tag{20}$$

$$w_e^{RS} = \frac{ab(2b + r) - \delta[(6b^2 - r^2)k_e + brk_o]}{2bmd(4b^2 - r^2)} + \frac{(6b^2 - r^2)(\beta c_e - \lambda\alpha e_o) + br\alpha(c_o + c_n e_o)}{2(4b^2 - r^2)}. \tag{21}$$

Proof of Proposition 5.1.

Taking the first-order derivative of the optimal delivery fees and retail prices with respect to k_o, we obtain the following results.

Under MS scenario, it holds that

$$\frac{\partial w_o^{MS}}{\partial k_o} = -\frac{4b\delta}{md(4b^2 - r^2)} < 0. \ \frac{\partial w_e^{MS}}{\partial k_o} = -\frac{2r\delta}{md(4b^2 - r^2)} < 0.$$
$$\frac{\partial p_o^{MS}}{\partial k_o} = -\frac{2b\delta}{4b^2 - r^2} < 0. \ \frac{\partial p_e^{MS}}{\partial k_o} = -\frac{r\delta}{4b^2 - r^2} < 0.$$

Under RS scenario, it holds that

$$\frac{\partial w_o^{RS}}{\partial k_o} = -\frac{(6b^2 - r^2)\delta}{2bmd(4b^2 - r^2)} < 0. \ \frac{\partial w_e^{RS}}{\partial k_o} = -\frac{r\delta}{2md(4b^2 - r^2)} < 0.$$
$$\frac{\partial p_o^{RS}}{\partial k_o} = -\frac{b\delta}{4b^2 - r^2} < 0. \ \frac{\partial p_e^{RS}}{\partial k_o} = -\frac{r\delta}{2(4b^2 - r^2)} < 0.$$

Taking the first-order derivative of the optimal delivery fees and retail prices with respect to k_e, we obtain the following results.

Under MS scenario, it holds that

$$\frac{\partial w_o^{MS}}{\partial k_e} = -\frac{2r\delta}{md(4b^2-r^2)} < 0. \ \frac{\partial w_e^{MS}}{\partial k_e} = -\frac{4b\delta}{md(4b^2-r^2)} < 0.$$
$$\frac{\partial p_o^{MS}}{\partial k_e} = -\frac{r\delta}{4b^2-r^2} < 0. \ \frac{\partial p_e^{MS}}{\partial k_e} = -\frac{2b\delta}{4b^2-r^2} < 0.$$

Under RS scenario, it holds that

$$\frac{\partial w_o^{RS}}{\partial k_e} = -\frac{r\delta}{2bmd(4b^2-r^2)} < 0. \ \frac{\partial w_e^{RS}}{\partial k_e} = -\frac{(6b^2-r^2)\delta}{2bmd(4b^2-r^2)} < 0.$$
$$\frac{\partial p_o^{RS}}{\partial k_e} = -\frac{r\delta}{2(4b^2-r^2)} < 0. \ \frac{\partial p_e^{RS}}{\partial k_e} = -\frac{b\delta}{4b^2-r^2} < 0.$$

Proof of Proposition 5.2.

Taking the first-order derivative of the optimal delivery fees and retail prices with respect to δ, we obtain the following results.

Under MS scenario, it holds that

$$\frac{\partial w_o^{MS}}{\partial \delta} = -\frac{2(rk_e+2bk_o)}{md(4b^2-r^2)} < 0. \ \frac{\partial w_e^{MS}}{\partial \delta} = -\frac{2(2bk_e+rk_o)}{md(4b^2-r^2)} < 0.$$
$$\frac{\partial p_o^{MS}}{\partial \delta} = -\frac{rk_e+2bk_o}{4b^2-r^2} < 0. \ \frac{\partial p_e^{MS}}{\partial \delta} = -\frac{2bk_e+rk_o}{4b^2-r^2} < 0.$$

Under RS scenario, it holds that

$$\frac{\partial w_o^{RS}}{\partial \delta} = -\frac{brk_e+(6b^2-r^2)k_o}{2bmd(4b^2-r^2)} < 0. \ \frac{\partial w_e^{RS}}{\partial \delta} = -\frac{(6b^2-r^2)k_e+brk_o}{2bmd(4b^2-r^2)} < 0.$$
$$\frac{\partial p_o^{RS}}{\partial \delta} = -\frac{rk_e+2bk_o}{2(4b^2-r^2)} < 0. \ \frac{\partial p_e^{RS}}{\partial \delta} = -\frac{2bk_e+rk_o}{4b^2-r^2} < 0.$$

Proof of Proposition 5.3.

Taking the first-order derivative of the optimal delivery fees and retail prices with respect to c_n, we obtain the following results.

Under MS scenario, it holds that

$$\frac{\partial w_o^{MS}}{\partial c_n} = \frac{2\alpha b^2 e_o}{4b^2-r^2} > 0. \ \frac{\partial w_e^{MS}}{\partial c_n} = \frac{br\alpha e_o}{4b^2-r^2} > 0.$$
$$\frac{\partial p_o^{MS}}{\partial c_n} = \frac{b^2 md\alpha e_o}{4b^2-r^2} > 0. \ \frac{\partial p_e^{MS}}{\partial c_n} = \frac{brmd\alpha e_o}{2(4b^2-r^2)} > 0.$$

Under RS scenario, it holds that

$$\frac{\partial w_o^{RS}}{\partial c_n} = \frac{(6b^2-r^2)\alpha e_o}{2(4b^2-r^2)} > 0. \ \frac{\partial w_e^{RS}}{\partial c_n} = \frac{br\alpha e_o}{2(4b^2-r^2)} > 0.$$
$$\frac{\partial p_o^{RS}}{\partial c_n} = \frac{b^2 md\alpha e_o}{4b^2-r^2} > 0. \ \frac{\partial p_e^{RS}}{\partial c_n} = \frac{bmdr\alpha e_o}{2(4b^2-r^2)} > 0.$$

Proof of Proposition 5.4.

Taking the first-order derivative of the optimal delivery fees and retail prices with respect to b, we obtain the following results.

Under MS scenario, it holds that

$$\begin{aligned}\frac{\partial w_o^{MS}}{\partial b} &= -\frac{4br}{(4b^2-r^2)^2 md}[2a + dmr\alpha(c_o - c_n e_o) - 4\delta k_e] \\ &\quad - \frac{4b^2+r^2}{(4b^2-r^2)^2 md}[2a + dmr(\beta c_e - \alpha\lambda e_o) - 4\delta k_o] \\ &\leq -\frac{4br}{(4b^2-r^2)^2 md}\{4a + dmr[(\beta c_e - \alpha\lambda e_o) + \alpha(c_o - c_n e_o)] - 4\delta(k_o + k_e)\} \\ &< 0.\end{aligned}$$

$$\begin{aligned}\frac{\partial w_e^{MS}}{\partial b} &= -\frac{4b^2+r^2}{(4b^2-r^2)^2 md}[2a + dmr\alpha(c_o - c_n e_o) - 4\delta k_e] \\ &\quad - \frac{4br}{(4b^2-r^2)^2 md}[2a + dmr(\beta c_e - \alpha\lambda e_o) - 4\delta k_o] \\ &\leq -\frac{4br}{(4b^2-r^2)^2 md}\{4a + dmr[(\beta c_e - \alpha\lambda e_o) + \alpha(c_o - c_n e_o)] - 4\delta(k_o + k_e)\} \\ &< 0.\end{aligned}$$

$$\begin{aligned}\frac{\partial p_o^{MS}}{\partial b} &= -\tfrac{a}{2(b-r)^2} - \tfrac{2br}{(4b^2-r^2)^2}[2a + dmr\alpha(c_o - c_n e_o) - 4\delta k_e] \\ &\quad - \tfrac{4b^2+r^2}{2(4b^2-r^2)^2}[2a + dmr(\beta c_e - \alpha\lambda e_o) - 4\delta k_o] \\ &< 0.\end{aligned}$$

$$\begin{aligned}\frac{\partial p_e^{MS}}{\partial b} &= -\tfrac{a}{2(b-r)^2} - \tfrac{4b^2+r^2}{2(4b^2-r^2)^2}[2a + dmr\alpha(c_o - c_n e_o) - 4\delta k_e] \\ &\quad - \tfrac{2br}{(4b^2-r^2)^2}[2a + dmr(\beta c_e - \alpha\lambda e_o) - 4\delta k_o] \\ &< 0.\end{aligned}$$

Under RS scenario, it holds that

$$\begin{aligned}\frac{\partial w_o^{RS}}{\partial b} &= -\tfrac{r^2(12b^2-r^2)\delta k_o}{2b^2dm(4b^2-r^2)^2} - \tfrac{2br}{dm(4b^2-r^2)^2}[2a + dmr\alpha(c_o - c_n e_o) - 2\delta k_e] \\ &\quad - \tfrac{4b^2+r^2}{2(4b^2-r^2)^2md}[2a + dmr(\beta c_e - \alpha\lambda e_o) - 6\delta k_o] \\ &< 0.\end{aligned}$$

$$\begin{aligned}\frac{\partial w_e^{RS}}{\partial b} &= -\tfrac{r^2(12b^2-r^2)\delta k_e}{2b^2dm(4b^2-r^2)^2} - \tfrac{2br}{dm(4b^2-r^2)^2}[2a + dmr(\beta c_e - \alpha\lambda e_o) - 2\delta k_o] \\ &\quad - \tfrac{4b^2+r^2}{2(4b^2-r^2)^2md}[2a + dmr\alpha(c_o - c_n e_o) - 6\delta k_e] \\ &< 0.\end{aligned}$$

$$\begin{aligned}\frac{\partial p_o^{RS}}{\partial b} &= -\tfrac{a}{2(b-r)^2} - \tfrac{2br}{(4b^2-r^2)^2}[2a + dmr\alpha(c_o - c_n e_o) - 2\delta k_e] \\ &\quad - \tfrac{4b^2+r^2}{2(4b^2-r^2)^2}[2a + dmr(\beta c_e - \alpha\lambda e_o) - 2\delta k_o] \\ &< 0.\end{aligned}$$

$$\begin{aligned}\frac{\partial p_e^{RS}}{\partial b} &= -\tfrac{a}{2(b-r)^2} - \tfrac{4b^2+r^2}{2(4b^2-r^2)^2}[2a + dmr\alpha(c_o - c_n e_o) - 2\delta k_e] \\ &\quad - \tfrac{2br}{(4b^2-r^2)^2}[2a + dmr(\beta c_e - \alpha\lambda e_o) - 2\delta k_o] \\ &< 0.\end{aligned}$$

Taking the first-order derivative of the optimal delivery fees and retail prices with respect to r, we obtain the following results.

Under MS scenario, it holds that

$$\begin{aligned}\frac{\partial w_o^{MS}}{\partial r} &= \tfrac{4br}{(4b^2-r^2)^2md}[a + dmr\alpha(c_o - c_n e_o) - 2\delta k_o] \\ &\quad + \tfrac{4b^2+r^2}{(4b^2-r^2)^2md}[a + dmr(\beta c_e - \alpha\lambda e_o) - 2\delta k_o] \\ &> 0.\end{aligned}$$

$$\begin{aligned}\frac{\partial w_e^{MS}}{\partial r} &= \tfrac{4b^2+r^2}{(4b^2-r^2)^2md}[a + dmr\alpha(c_o - c_n e_o) - 2\delta k_e] \\ &\quad + \tfrac{4br}{(4b^2-r^2)^2md}[a + dmr(\beta c_e - \alpha\lambda e_o) - 2\delta k_o] \\ &> 0.\end{aligned}$$

$$\begin{aligned}\frac{\partial p_o^{MS}}{\partial r} &= \tfrac{a}{(b-r)^2} + \tfrac{4br}{(4b^2-r^2)^2}[a + bdm\alpha(c_o - c_n e_o) - 2\delta k_o] \\ &\quad + \tfrac{4b^2+r^2}{(4b^2-r^2)^2}[a + bdm(\beta c_e - \alpha\lambda e_o) - 2\delta k_e] \\ &> 0.\end{aligned}$$

$$\begin{aligned}\frac{\partial p_e^{MS}}{\partial r} &= \tfrac{a}{(b-r)^2} + \tfrac{4b^2+r^2}{(4b^2-r^2)^2}[a + bdm\alpha(c_o - c_n e_o) - 2\delta k_o] \\ &\quad + \tfrac{4br}{(4b^2-r^2)^2}[a + bdm(\beta c_e - \alpha\lambda e_o) - 2\delta k_e] \\ &> 0.\end{aligned}$$

Under RS scenario, it holds that

$$\frac{\partial w_o^{RS}}{\partial r} = \frac{2br}{dm(4b^2-r^2)^2}[a + bdmr\alpha(c_o - c_n e_o) - \delta k_o] + \frac{4b^2+r^2}{2(4b^2-r^2)^2 md}[a + bdm(\beta c_e - \alpha\lambda e_o) - \delta k_o] > 0.$$

$$\frac{\partial w_e^{RS}}{\partial r} = \frac{2br}{dm(4b^2-r^2)^2}[a + bdm(\beta c_e - \alpha\lambda e_o) - \delta k_o] + \frac{4b^2+r^2}{2(4b^2-r^2)^2 md}[a + bdmr\alpha(c_o - c_n e_o) - \delta k_o] > 0.$$

$$\frac{\partial p_o^{RS}}{\partial r} = \frac{a}{(b-r)^2} + \frac{4br}{(4b^2-r^2)^2}[a + bdm\alpha(c_o - c_n e_o) - \delta k_o] + \frac{4b^2+r^2}{(4b^2-r^2)^2}[a + bdm(\beta c_e - \alpha\lambda e_o) - \delta k_e] > 0.$$

$$\frac{\partial p_e^{RS}}{\partial r} = -\frac{a}{(b-r)^2} + \frac{4b^2+r^2}{(4b^2-r^2)^2}[a + bdm\alpha(c_o - c_n e_o) - \delta k_o] - \frac{4br}{(4b^2-r^2)^2}[a + bdm(\beta c_e - \alpha\lambda e_o) - \delta k_e] > 0.$$

Proof of Proposition 6.1.

Recalling the Eqs. (10), (11), (18) and (19), we can obtain that

$$w_o^{RS} - w_o^{MS} = -\frac{r^2(a-2\delta k_o)}{2bdm(4b^2-r^2)} - \frac{r[a+bdm(\beta c_e-\alpha\lambda e_o)-3\delta k_e]}{2dm(4b^2-r^2)} - \frac{(2b^2-r^2)[a+bdm\alpha(c_o-c_n e_o)-\delta k_o]}{2bdm(4b^2-r^2)} < 0.$$

$$w_e^{RS} - w_e^{MS} = -\frac{r^2(a-2\delta k_e)}{2bdm(4b^2-r^2)} - \frac{r[a+bdm(\beta c_e-\alpha\lambda e_o)-3\delta k_o]}{2dm(4b^2-r^2)} - \frac{(2b^2-r^2)[a+bdm\alpha(c_o-c_n e_o)-\delta k_e]}{2bdm(4b^2-r^2)} < 0.$$

Recalling the Eqs. (12), (13), (20) and (21), we can obtain that

$$p_o^{RS} - p_o^{MS} = \frac{\delta(rk_e+2bk_o)}{2(4b^2-r^2)} > 0.$$

$$p_e^{RS} - p_e^{MS} = \frac{\delta(rk_o+2bk_e)}{2(4b^2-r^2)} > 0.$$
